# As long as (I think) my husband agrees…: role of perceived partner approval in contraceptive use among couples living in military camps in Kinshasa, DRC

**DOI:** 10.1186/s12978-021-01256-y

**Published:** 2022-01-12

**Authors:** Julie H. Hernandez, Saleh Babazadeh, Philip A. Anglewicz, Pierre Z. Akilimali

**Affiliations:** 1 Department of International Health and Sustainable Development, Tulane University School of Public Health and Tropical Medicine, 1440 Canal St, New Orleans, LA 70112 USA; 2 Department of Health Policy and Management, Tulane University School of Public Health and Tropical Medicine, New Orleans, LA USA; 3 Department of Population, Family, and Reproductive Health, Bill & Melinda Gates Institute for Population and Reproductive Health, Johns Hopkins Bloomberg School of Public Health, Baltimore, MD USA; 4 Kinshasa School of Public Health, University of Kinshasa, Kinshasa, Democratic Republic of Congo

**Keywords:** Contraception, Men, Partner approval, Couple communication, Sub-Saharan Africa

## Abstract

**Background:**

Male partner’s approval is a key determinant of contraceptive use for women living in Sub-Saharan Africa and improving men’s support and couple communication is a cornerstone of family planning programs. However, approval is often only measured through the women’s perception of their partner’s opinion.

**Methods:**

This study conducted in Kinshasa compares contraceptive approval variables from matched male and female partners (n = 252 couples) to establish the frequency of (in)accurate perceptions by the woman, then test their association with modern contraceptive use. Additional regressions estimate individual and couple variables associated with (in)correct perceptions.

**Results:**

Results confirm women are poorly aware of their partner’s opinion but indicate that perceived approval or disapproval by the woman is a much stronger determinant of modern contraceptive use than her partner’s actual opinion. Higher educational achievement from the woman is the strongest driver of misunderstanding her partner’s approval.

**Conclusions:**

Women’s perceptions of partner’s approval are much stronger determinant of contraceptive use than the latter’s actual opinion, and stereotyping men’s opinion of family planning is a common error of appreciation. However, findings also suggest these misunderstandings might serve women’s capacity to negotiate contraceptive use.

**Supplementary Information:**

The online version contains supplementary material available at 10.1186/s12978-021-01256-y.

## Plain language summary

Research indicates that women living in Sub-Saharan Africa may not use contraceptive methods if their partner disapproves. However, there are methodological gaps in how this relation has been measured so far. For example, women are often the only ones asked whether their partner approves of contraception and surveys rarely assess how women know of their partner’s disapproval and how strongly it has been communicated to them, nor do they ask said partner for his actual opinion on the matter.

In this study we address some of those questions by interviewing men and women from married couples separately and comparing their opinion of family planning use. The research uses a population-based survey conducted among couples living in military camps in the capital city of the Democratic Republic Congo, Kinshasa.

The results show that women overall are poorly aware of their partner’s actual opinion, but act based on those perceptions, nonetheless. In particular, women whose husband disapproves of family planning but (falsely) perceive his approval have some of the highest odds in our cohort for contraceptive use. Conversely, women in a “false negative” scenario (husband approves but they perceive disapproval) are less likely to use modern contraception. Additional analysis indicates that this latter scenario is more common among women who are more educated than their partner, possibly because they are stereotyping his family planning desires. The findings and the discussion also raise the possibility that women may however benefit from ignoring their partners’ true wishes in order to fulfill their own contraceptive choice.

**Supplementary Information:**

The online version contains supplementary material available at 10.1186/s12978-021-01256-y.

## Résumé

**Contexte:**

L’approbation du conjoint est un facteur clé de l’utilisation des contraceptifs chez les femmes vivant en Afrique subsaharienne, et l’amélioration du soutien des hommes et de la communication au sein du couple est une pierre angulaire des programmes de planification familiale. Cependant, l’approbation conjugale n’est souvent mesurée qu’à travers la perception des femmes de ce que pensent leurs partenaires.

**Méthodes:**

Cette étude menée à Kinshasa compare les variables d’approbation de l’utilisation contraceptive entre les hommes et les femmes formant un couple (n = 252 couples) pour établir la fréquence des perceptions (in)exactes des femmes, puis teste leur association à l’utilisation contraceptive moderne. Des régressions additionnelles permettent d’estimer les variables individuelles et des couples associées à ces perceptions (in)exactes.

**Résultats:**

Les résultats confirment que les femmes ont une faible connaissance de l’opinion de leurs partenaires quant à l’utilisation contraceptive, mais indiquent que la perception par ces femmes d’un avis favorable ou défavorable est un déterminant bien plus fort de l’utilisation contraceptive moderne que l’opinion réelle des partenaires. Le niveau d’études plus élevé de la femme est le facteur le plus largement associé à une perception erronée de l’avis de son partenaire.

**Conclusions:**

Les perceptions des femmes de l’avis de leurs conjoints sont un déterminant bien plus fort de l’utilisation contraceptive que l’opinion réelle de ces derniers, et stéréotyper l’opinion des hommes concernant la planification familiale est une erreur d’appréciation courante. Cependant, les résultats de recherche suggèrent aussi que ces malentendus pourraient servir la capacité des femmes à négocier leur utilisation de la contraception.

## Background

Men’s involvement in supporting contraceptive decision and contraceptive use has been a leitmotiv of family planning programs since the 1994 International Conference on Population and Development [1], with strategies ranging from changing men’s fertility aspirations and attitude towards contraceptive use [2], increasing women’s agency in fertility related decision-making [3] and improving couple communication [4–6]. In Sub-Saharan Africa, the assumptions that men hold “traditional” desires for large families and that they play a dominant role in decision-making in the household positions them as key players in interventions designed to improve women’s contraceptive use [7]. Indeed, research consistently finds that male approval is associated with increased contraceptive use independently of other factors such as women’s sense of self-efficacy or perceived contraceptive accessibility [8–12]. Furthermore, the effect appears to be bi-directional since men’s disapproval stand in some studies as the single more powerful deterrent towards use of family planning. [13–15]

The key role of male partner’s approval for contraceptive use is however complicated because existing evidence also strongly suggest that women tend to be inaccurate in the perception of their partner’s actual opinion regarding their use of family planning [16]. The low validity of wives’ report of husband’s approval of family planning has been attributed to lack of intra-couple communication [17] and a tendency to stereotype their partner’s fertility desire (“All men want large families”) [18]. On the male partners’ side, the widely held belief that men should have the final say in family matters, but women are nonetheless solely responsible for managing their pregnancies [19], further deepens the possibilities for misunderstandings. Existing studies comparing men’s and women’s fertility desire indicate that most of these misunderstanding arise because respondents typically underestimate their partner’s desire to limit the family size [15, 18], or because women conflate their partner’s desire for more children with their opposition to contraceptive use [20]. As a result, a trend towards “false negative” scenarios, where women perceive disapproval from their partners, but surveys among men suggest that a majority of them are supportive of contraceptive use has been noted, including in high fertility contexts such as Nigeria and Ethiopia [21, 22]

Few studies are available to compare female perception of approval and men’s actual opinion of contraceptive use, and the sole availability of perceived partner preference rather than true partner preference as an explanatory variable if often cited as a limitation [12, 17, 23]. The research presented here provides insights on these misunderstandings regarding contraceptive use by comparing matched male and female partners’ independent answers to the same questions. The couples were all recruited within military camps dispersed throughout Kinshasa, the capital city of the Democratic Republic of Congo (DRC). The DRC, one of the poorest countries in the world, also has one of the world’s highest fertility rates (6.0) and lowest modern contraceptive prevalence (14.2% on average in the country and 24.5% in Kinshasa) [24, 25]. As an FP2020 focus country, the DRC and particularly the provinces of Kinshasa and Kongo Central, are regularly targeted by population surveys, and in 2016 the military authorities requested a specific iteration of the PMA2020 (Performance in Monitoring and Accountability) survey to be implemented in military camps throughout Kinshasa to assess whether those populations had specific need, compared to the general population living in surrounding neighborhoods. The smaller scope of the military study allowed for researchers to add specific modules on topics which were of interest across the DRC general population but could typically not be integrated into the global PMA survey. Results from the first round of survey indicated among other findings that women residing in military camps had high unmet need for family planning services [26]. The data presented in this article comes from the second round of PMA survey in military camps, implemented in January 2020. This round intended to capture contraceptive trends after four years of various interventions lead by family planning partners in military camps, but also included matching questions about fertility desires and approval of contraceptive use that were independently asked from male and female partners in established couples. The objective of the analyses presented here are first to estimate the prevalence of correct perception, false positive and false negative perceptions of men’s approval of contraceptive use by their female partner, and to compare the relative importance of actual and perceived approval with regards to women’s contraceptive use. We then seek to determine individual and couple characteristics associated with correct and incorrect perception of male partner’s approval by the woman, with a specific attention to false negative and false positive scenarios.

## Methods

### Data

Data collection within the population of military camps used a multi-stage cluster design approach. There are 17 military camps in Kinshasa, which are home to an estimated 3% (305,000) of the total population of the city [26]. Households in these camps tend to have at least one person in active service duty, however extended families with no direct army affiliation can also reside in the camps. All residents however have access to military health facilities within the camp, including family planning services offered by the Military Program for Reproductive Health (PMSR).

Out of the 17 military camps in Kinshasa, 10 were randomly drawn, proportionate to population size. These 10 military camps were then divided into enumeration areas (EAs) of similar size. One EA then was randomly selected in each of the 10 camps. The data collection team consisted of female resident enumerators (REs). All REs were themselves residents of the military camps, had been trained in technical and ethical aspects of the survey and had participated in the previous round of PMA survey in 2016. They first listed all the households in the selected EA. As in 2016 33 households were randomly selected in each EA. Since we were interested in interviewing couples, we only interviewed women who were married or in union and currently living with their partners in those households. Women who were divorced, widowed or have never been married (about 2% of the total sample) were excluded from our analysis. In order to reach a sample size comparable to the one we used in 2016 while limiting our population to women married or in union, we randomly added 14 households in each RA for a total of 47. Since the sample size was calculated based on the women, women in selected household were interviewed first, followed by their male partners. Although some men indicated being in a polygamous relationship, no two women in our sample were linked to the same partner. Response rate for the survey was above 99%. Respondents for each couple were interviewed by the same RE but independently from each other, at a location of their choosing and in separate rooms to guarantee privacy and confidentiality. Interviews were conducted in French or any local dialect the respondents were most comfortable with (most often Lingala which is the *lingua franca* of the DRC military) and recorded in French in smartphones running the OpenDataKit application. [26]

The data collection and research protocol were approved by the Tulane University IRB (study 492318) and the Kinshasa School of Public Health ethics committee (#ESP/CE/070/2017).

All participating individuals provided written and informed consent to take part in this study.

### Analysis

This study focuses on two sets of outcome variables (1) correct or incorrect perceptions of male partner’s approval of contraceptive use among female respondents, and (2) modern contraceptive use among female respondents. Perception of male partner’s approval was defined by the woman’s response to the question “Do you believe your partner approves / would approve of your use of modern contraceptives?” Possible answers included “yes”, “no”, “depends on the method”, “do not know”, for which we categorized “yes” as “perceived approval”, while all other were grouped as “perceived disapproval”. The same binary categories were applied to the male respondents’ responses to the question: “would you approve if your partner wanted to use contraception?” (Interviewers did not disclose to the man whether his female partner had indicated using contraception). Any other response than “yes” was recorded as disapproval. Comparing women’s perceptions and men’s actual opinions led to four main scenarios: Women correctly perceive approval (S1); Women correctly perceives disapproval (S2), women incorrectly perceive disapproval (false negative) (S3), and women incorrectly perceive approval (false positive) (S4), combinations of which led to “correct perception of partner’s opinion” (S1 + S2 = S5) and “incorrect perception” (S3 + S4 = S6).

We first conducted bivariate analysis to test the association of those scenarios with modern contraceptive use by the couple; then to test the association of modern contraceptive use and mentioned scenarios after controlling for other influential independent variables, we included individual and couples’ characteristics in a multivariable regression model.

Finally, we conducted another set of regression analysis to determine whether certain individual or couple characteristics were significantly associated with perception scenarios we found likely to increase or decrease contraceptive use, including incorrect perception of partner’s opinion, perceived approval, and false negative perception. Independent variables included in all regressions were factors assumed to influence power relations within the couple and contraceptive use, such as woman’s age, individual education and educational difference within couple, number of living children, whether each partner wants more children, whether each partner has discussed his or her ideal number of children, the man’s ideal family size and his military status (active military or not).

## Results

The study sample included 252 couples, married or living in union. As shown in Table 1, women were on average younger and slightly less educated than men. About half of the men and only 2.9% of the women were active military members. Most individuals had completed at least secondary education (83.2% of women and 90.3% of men) and educational achievements were similar within 57.0% of couples, whereas a quarter of men (25.9%) were more educated and 17.1% of women were more educated than their partner (See Table 1). Observed discrepancies in marital status, with men disproportionately reporting being married and with a slightly higher number of living children most likely come from the fact that their live-in partner in the camp is not always their official or primary spouse. Regardless, all family planning questions were asked in relation to said live-in partner, who was also the person interviewed as the other half of the couple.

**Table 1:** Demographic characteristics of the couples in the study (N = 252)

	Women (%)	Men (%)
Age		
< 20	8.6	1.2
20–24	15.6	4.9
25–29	18.2	7.9
30–34	20.9	15.3
35–39	16.0	15.2
> = 40	20.7	55.5
Education		
Never/primary	16.8	9.7
Secondary	64.6	68.8
Higher	18.6	21.5
Education difference		
Same	57.0	n/a
Man has more education	25.9	n/a
Woman has more education	17.1	n/a
Active military service	2.9	56.2
Married	45.8	73.2
In union	54.0	26.8
Mean number of living children	2.5	2.9

In Table 2, we compare male partner’s approval of contraceptive use with women’s perception of their partner’s approval. When asked separately, 59.8% of men stated they disapproved of their female partners using contraception, but a larger proportion of women (73.4%) perceived disapproval. When women were mistaken in their perception of their partner’s approval, it was more likely that they perceived that he disapproved when he in fact approved, instead of vice-versa: a quarter (24.9%) of women erroneously thought their partner disapproved of contraceptive use (false negative). On the other hand, 11.3% of women stated their partner approved of contraceptive use, when he in fact did not (false positive). Overall, the woman’s perception of their partner (dis)approval was correct 63.8% of the time (Table 2).

**Table 2:** Percentage of women who correctly or incorrectly perceive approval and disapproval

		Male partner’s approval		
		Approves (N)	Disapproves (N)	Total (N)
Female partner’s perception	Perceives approval	15.3% (47) *S1*	11.3% (32) *S4*	26.6% (79)
	Perceives disapproval	24.9% (61) *S3*	48.5% (112) *S2*	73.4% (173)
	Total	40.2% (108)	59.8% (144)	100.0% (252)

We then looked at the association between the scenarios in Table 2 (i.e., the combination of partner’s actual opinion and woman’s perception of her partner’s approval or disapproval) and modern contraceptive use by the couple (Table 3). As would be expected, modern contraceptive use is highest (41.3%) when the man approves of it and his female partner correctly perceives his approval and is lowest (19.4%) when the woman correctly perceives her partner’s disapproval (p = 0.1201).

**Table 3:** Association between perception of partner's approval and modern contraceptive use

	Freq	Modern contraceptive use (%)	P-value*
Male partner approves/wife perceives approval (S1)	47	41.3	0.0828
*Wife perceives approval* *(Regardless of partner’s true opinion*)	79	40.2	0.0116
Male partner disapproves/wife perceives approval (False positive S4)	32	38.7	0.0730
Male partner approves (regardless of wife’s perception)	108	27.9	0.5541
Male partner disapproves (regardless of wife’s perception)	144	23.1	0.5541
Male partner agrees/ Wife perceives disapproval (False negative S3)	61	19.6	0.1672
*Wife perceives disapproval* *(Regardless of male partner’s true opinion)*	173	19.5	0.0116
Male partner disagrees/wife perceives disagreement (S2)	112	19.4	0.1201

Our results however indicate that modern contraceptive use is similarly high and low regardless of the man’s actual opinion, provided that the female partner perceives it as approval or disapproval, and the associations are much more significant. Modern contraceptive use is 40.3% (p-value < 0.05) for women who believe their partner approve of it, and 19.5% (p-value < 0.05) for those who believe their partner disapproves. In fact, 38.7% of women who incorrectly perceive their partner’s approval (false positive) use modern contraception (p < 0.1), only 2.6 percentage points less than women who correctly believe their partner’s support their use of a modern method. At the other end of the spectrum, women who incorrectly perceive their partner’s disapproval (false negative) are among the lowest users of modern contraception, although the relationship is not significant.

To further test the association of approval/perception scenarios with modern contraceptive use after controlling for socio-demographic characteristics of men and women, we performed a multivariate logistic regression on modern contraceptive use. Results from this analysis confirm the much more significant role of the woman’s perception of her partner’s approval as a determinant of contraceptive use (see Table 4). When their partner disapproved of contraceptive use, women who incorrectly perceived approval had 2.52 times greater odds of using a modern method than women who correctly read their partner’s opinion, and the relationship was highly significant (p-value = 0.007). Conversely women who erroneously perceived their partner’s disapproval (false negative) appeared slightly less likely to use modern contraception, but the relationship was not significant (OR = 0.92, p-value = 0.805).

**Table 4:** Association of individual and couple variables with modern contraceptive use

Modern contraceptive use (0,1)	Odds ratio	SE	P-value
Actual and perceived approval of contraceptive use			
Husband disagrees/wife perceive disagreement (S2)	Ref		
Husband approves/wife perceive approval (S1)	1.93	1.04	0.256
Husband disapproves/wife perceive approval (S4)	2.52***	0.67	0.007
Husband agrees/wife perceive disagreement (S3)	0.92	0.31	0.805
Woman's age			
< 20	Ref		
20–24	1.59	0.66	0.295
25–29	1.58	0.48	0.165
30–34	0.76	0.44	0.644
35–39	0.53	0.24	0.193
> = 40	0.21**	0.12	0.027
Educational difference			
Same education	Ref		
Man has more education	1.16	0.49	0.728
Woman has more education	0.89	0.29	0.722
Number of living children			
0–1	Ref		
2–4	1.10	0.71	0.887
> 4	3.11**	1.52	0.045
Man is in active military service			
No	Ref		
Yes	0.44*	0.19	0.092
Man wants more children			
No			
Yes	0.79	0.46	0.699
Woman wants more children			
No			
Yes	1.14	0.48	0.773
Man discussed ideal number of children with his partner			
No	Ref		
Yes	1.28	0.27	0.273
Woman discussed ideal number of children with her partner			
No	Ref		
Yes	0.75	0.33	0.536
Man's ideal size of family	1.05	0.06	0.389

Among other individual characteristics of women and men, few were significantly associated with modern contraceptive use: odds ratio for contraceptive use declined with age and women over 40 were significantly less likely to use a modern method (OR = 0.21, p-value < 0.05). Women whose partner was in active military were also significantly less likely to use a modern method (OR = 0.44, p-value < 0.1). On the other hand, women who had four or more living children were three times more likely to use a modern method (OR = 3.01, p-value < 0.05). Differences in educational status, fertility desire variables such as either partner wanting more children and ideal family size or having discussed the desired number of children were not significantly associated with modern contraceptive use.

Since perception of their partner’s approval is so significantly associated with contraceptive use by the women, it seems important to determine whether specific individual and couple profiles were associated with (in)correct perceptions, as well as false positive and false negative perceptions. To achieve that goal, we introduced three logistic regression models (see Table 5) testing the associations between women and couples’ characteristics and three specific scenarios. We chose the scenarios based on the strong association found in the previously shown model (Table 4): (1) woman incorrectly perceives her partner’s opinion (36.2% of our sample), (2) woman perceives approval regardless of her partner’s opinion (26.6% of our sample), and (3) woman incorrectly perceives disapproval on her partner’s part (false negative = 24.9% of our sample). Because only a small number of women fell into the false positive category, we did not perform a multivariate regression analysis for this outcome.

**Table 5:** Multivariate logistic regression analysis of correct and incorrect perceptions of partner's approval of contraceptive use

	Incorrect perception	Perceives approval	Incorrect perception of disapproval (false negative)
Woman's age			
< 20	Ref		
20–24	2.03	0.69	3.57
25–29	0.52	0.27	1.40
30–34	1.39	0.15*	5.72
35–39	0.79	0.13	2.20
> = 40	0.72	0.08	2.01
Education difference			
Same education	Ref		
Man has more education	1.64	1.73*	1.14
Woman has more education	1.91**	0.35**	2.84***
Number of living children			
0–1	Ref		
2–4	1.90	2.81**	1.46
> 4	1.25	6.62**	1.03
Man is active military service			
No	Ref		
Yes	1.51	0.58	1.65
Man wants more children			
No	Ref		
Yes	0.84	0.45	1.50
Woman wants more children			
No	Ref		
Yes	1.15	2.13**	0.95
Man discussed ideal number of children with his partner			
No	Ref		
Yes	0.56	4.67*	0.27***
Woman discussed ideal number of children with his partner			
No	Ref		
Yes	0.67	0.73	0.74
Man's ideal size of family	0.93	0.99	0.92

The results of this regression indicate that among all characteristics included in the model, number of living children was strongly associated with perception of approval for contraceptive use: women with 2 to 4 children and women with more than 4 children were twice to five times more likely to declare that their partner approved of their contraceptive use (OR = 2.81 and 6.62 respectively, p-value < 0.05). The same was true of women who stated that they wanted more children (OR = 2.13, p-value < 0.05).

On the other hand, educational differences were negatively associated with perceiving one’s partner’s approval: women who were more educated than their husband significantly less likely to perceive approval (OR = 0.35, p-value < 0.05). This last variable was also significantly associated with incorrect perception of their partner approval (OR = 1.9 p-value < 0.05) and even more so with incorrect perception of his disapproval: women who were more educated than their partner were almost three times more likely to fall in a “false negative” scenario (OR = 2.84, p-value < 0.01).

Having discussed the number of desired children made women less likely to hold an incorrect perception of their partner’s approval (OR = 0.69, p-value < 0.1), and the likelihood of a “false negative” decreased significantly when the man had discussed his desired number of children (OR = 0.27, p-value < 0.01).

Difference between man and woman ideal family size, ever or current use of modern contraception were initially added to the model but did not yield any meaningful results.

Since differences in educational attainment and having discussed desired number of children were strongly associated with (in-)correct perception of partner’s approval of contraceptive use, we ran a separate analysis to assess whether educational level was associated with increased communication about fertility desires (Table 6). While higher level of individual education for either partner was associated with the likelihood of having discussed the desired number of children (p-value < 0.1), educational difference did not have a significant bearing on this outcome. Thus, women who were more educated than their partner were not more or less likely to be more informed about his actual fertility desires.

**Table 6:** Association between individual education level and educational difference and likelihood of having discussed desired number of children

	Male discussed ideal number of children (%)	
Male education		P = 0.0883*
Never/primary	32.56	
Secondary	56.63	
Tertiary	74.94	

	Female discussed ideal number of children (%)	
Female education		P = 0.0815*
Never/primary	57.26	
Secondary	61.66	
Tertiary	74.25	

	Male discussed ideal number of children	
Education difference		P = 0.4641*
Same	62.88	
Man is more educated	58.9	
Woman is more educated	41.8	

	Female ideal number of children	
Education difference		P = 0.1990*
Same	64.36	
Man is more educated	57.35	
Woman is more educated	68.57	

## Discussion

Findings from this research among military couples residing in Kinshasa confirm that women tend to be poorly aware of partners’ opinion regarding contraceptive use: almost half of the female respondents in our sample were incorrect in their perception, with the majority incorrectly perceiving their partner’s disapproval (false negative), while about one in ten women was a false positive (erroneously perceiving approval from her partner). The false negative scenario, which concerned 24.9% of our sample, may be a product of stereotyping in the absence of spousal discussion regarding family planning, as observed in other high-fertility, low contraceptive use environments, where women tend to project on their partner the assumed attitude of most men living in those environments [11, 21],

These errors in assessing men’s opinion regarding contraception are concerning because our results indicate that, regardless of men’s actual approval of family planning use, it is their partner’s perception of that opinion that acts as a determinant of (non)-use, thus confirming findings from previous research conducted in Sub-Saharan Africa [11, 27]. When introducing individual and couple variables in the models, only a few expected characteristics remained as independent predictors of contraceptive use (number of living children) or non-use (women older than 40, male partner in the army), but perceived approval, especially in the case of false positive, remained a strong independent predictor of contraceptive use. Therefore, with nearly a quarter of all female participants holding a “false negative” perception, improving couple communication on the subject might increase contraceptive use if the male partner’s approval was more clearly shared.

Further analysis looking at variables associated with incorrect perception, perception of approval and “false negative” perception of partner’s opinion confirmed the potential importance of communication: women were four times more likely to perceive approval and four times less likely to hold a false negative perception if their partner had discussed the number of children they wanted. However, the relationship did not apply if only the woman had expressed her fertility desires, which confirms the weight of men’s preferences in fertility decisions [28]. Interestingly, women who had a large number of living children and those who stated that they wanted more children were also more likely to perceive approval from their partner to use contraception. This suggests that women who have fulfilled their or their partner’s fertility expectations, or at least who intend to do so, may feel they have earned the right to rest and thus assume they have their partner’s approval to use contraception for spacing their next pregnancies.

Aside from individual and joint fertility desires and communication, the fact that the likelihood of perceiving approval for family planning increased when men were more educated than their partner compounds existing evidence that increase in male education plays an important role in improving spousal communication and favorable attitudes towards family planning [29, 30]. On the other hand, women education, and particularly differences in education with their partner, played in a somewhat counter-intuitive ways in our results. While more educated women were more likely to have discussed their desired number of children, women who were more educated than their partners were also significantly more likely to be incorrect about his approval of contraceptive use and this error leaned heavily towards falsely perceiving disapproval. This could be because women with partners less educated than themselves are more prone to stereotyping their partner’s attitudes. But another possibility lies in more educated women knowingly misrepresenting their partner’s opposition to justify not using contraceptives. Whether they personally have high fertility desires, fear modern contraceptive side-effects, or hold other adverse beliefs towards contraception, more educated women are more likely to understand that partner’s opposition is a more acceptable response, because it shifts the responsibility away from them, when asked about their reasons for non-use. Research conducted in Sub-Saharan Africa consistently indicates that women tend to have more conservative attitudes than men [27], and that the classic inverse relationship between women’s education and lower demand for children is not as strongly marked as in other developing contexts [30].

This hypothesis further suggests that there might be some usefulness to the noted discrepancies in awareness of partner’s opinion regarding contraceptive use. Women who for any reason do not want to use modern contraceptive or prefer to have more children can outsource their agency in the matter in a more socially acceptable way by assigning disapproval of family planning to their husband. Conversely, women may feign ignorance of their partner’s disapproval (false positive) in order to avoid the accusation of covert use. Considering that most men in our sample still disapprove of contraceptive use and considering the weight of male perceived opinion in family planning decision, it would thus not always maximize benefits from the woman’s perspective to improve her awareness of that opinion. Wolff, Blanc et al., in their analysis of the role of couple negotiation in unmet need for contraception in Uganda, similarly pointed out that “where serious disagreement exists, increase communication may not be a universal good” [18], and other studies have highlighted the strain in gender relations, with occasionally brutal backlash on the women’s health agenda, created by interventions designed to frontally involve men in contraceptive decisions without considering cultural parameters first [31].

This study of discordance in actual and perceived approval of contraceptive use within couples thus teases an important issue for family planning programs as they strive to improve male engagement while maintaining women’s agency and rights-based family planning choices: whether women are honestly or purposefully mistaken about their partner’s approval of contraceptive use, these misunderstandings might on occasion better serve the woman’s particular contraceptive agenda than would perfect communication of family planning decisions in the couple [32].

Our study has several limitations however, particularly when it comes to desirability bias. Male respondents in particular may have been more likely to declare that they approved of their partner’s contraceptive use because they knew it was the “correct” answer. In addition, we have used “ever discussed the desired number of children” has a proxy for communication about family planning but it is unclear whether agreement on fertility desires accurately translates into agreement regarding contraceptive use within the couple. Besides, our survey instrument did not permit us to establish the frequency, length and quality of these conversations, all variables we would expect to have an impact on the woman’s perception of her partner’s opinion. We also chose modern contraceptive use, rather than intention to use, as a more reliably reported key outcome but this choice relies on the assumption that women living in military camps all face similar barriers and opportunities for accessing contraceptive services. Finally, future research would need to explore whether the military environment of the study affected our findings, and their generalizability to other couples living in Kinshasa, particularly as higher unmet need for family planning and more unequal gender relations in this context than in the general population have already been recorded [26, 33] and recent research indicates while similar contraceptive trends appear in both the general and the military populations in Kinshasa, the latter continue to exhibit certain specificities due to high mobility, particularly of male partners [34].

## Conclusion

This study provided strong evidence that regardless of men’s actual opinion on family planning, the perception women have of their approval or disapproval has a much stronger bearing on contraceptive use. While misunderstandings by the woman remain frequent, particularly when she is more educated than her partner, the false negative and false positive perceptions may have their own utility in family planning negotiations. As future research and programs search for effective ways to further engage men in supporting contraceptive use, our finding suggests that, considering the typical power imbalances in reproductive agency, absolute transparency in couple communication may also reduce the woman’s ability to negotiate her own fertility choices.

## Supplementary Information

**Additional file 1**. French language version of the article.

## Data Availability

The datasets generated and/or analyzed for this study are not publicly available due to privacy and confidentiality issues but are available from the corresponding author on reasonable request.

## Abbreviations

DRC: Democratic Republic of Congo
EA: Enumeration area
FP2020: Family planning 2020
ICPD: International Conference on Population and Development
PMA: Performance in monitoring and accounting
PMSR: Military Program for Reproductive Health
RE: Resident enumerator
