# Supplementary material for: As long as (I think) my husband agrees…: role of perceived partner approval in contraceptive use among couples living in military camps in Kinshasa, DRC
Source: Reprod Health. 2022 Jan 12;19:6. doi: 10.1186/s12978-021-01256-y (PMC8756630; doi:10.1186/s12978-021-01256-y)
Supplement: Supplementary file 1 — Additional file 1. French language version of the article. [file 12978_2021_1256_MOESM1_ESM.docx]

### Titre : Du moment que (je pense que) mon mari est d’accord… : le rôle de l’approbation perçue du conjoint dans l’utilisation de la contraception chez les couples vivant dans les camps militaires de Kinshasa, en RDC

Julie H. Hernandez**, Département de santé internationale et de développement durable, École de santé publique et de médecine tropicale de l’Université de Tulane, Nouvelle-Orléans, Louisiane, États-Unis.

Saleh Babazadeh, Département de politique et gestion de programmes sanitaires, École de santé publique et de médecine tropicale de l’Université de Tulane, Nouvelle-Orléans, Louisiane, États-Unis.

Philip A. Anglewicz, Institut Bill & Melinda Gates pour la population et la santé de la reproduction, Département de population, famille et santé reproductive, École de santé publique Bloomberg de l’Université de Johns Hopkins, Baltimore, Maryland, États-Unis.

Pierre Z. Akilimali, École de santé publique de Kinshasa, Université de Kinshasa, République Démocratique du Congo.

** Auteur référent: School of Public Health and Tropical Medicine, Tulane, 1440 Canal St, New Orleans, LA 70112

## Du moment que (je pense que) mon mari est d’accord… : le rôle de l’approbation perçue du conjoint dans l’utilisation de la contraception chez les couples vivant dans les camps militaires de Kinshasa, RDC

## **Résumé**

**Contexte :** L’approbation du conjoint est un facteur clé de l’utilisation des contraceptifs chez les femmes vivant en Afrique subsaharienne, et l’amélioration du soutien des hommes et de la communication au sein du couple est une pierre angulaire des programmes de planification familiale. Cependant, l’approbation conjugale n’est souvent mesurée qu’à travers la perception des femmes de ce que pensent leurs partenaires.

**Méthodes :** Cette étude menée à Kinshasa compare les variables d’approbation de l’utilisation contraceptive entre les hommes et les femmes formant un couple (n = 252 couples) pour établir la fréquence des perceptions (in)exactes des femmes, puis teste leur association à l’utilisation contraceptive moderne. Des régressions additionnelles permettent d’estimer les variables individuelles et des couples associées à ces perceptions (in)exactes.

**Résultats :** Les résultats confirment que les femmes ont une faible connaissance de l’opinion de leurs partenaires quant à l’utilisation contraceptive, mais indiquent que la perception par ces femmes d’un avis favorable ou défavorable est un déterminant bien plus fort de l’utilisation contraceptive moderne que l’opinion réelle des partenaires. Le niveau d’études plus élevé de la femme est le facteur le plus largement associé à une perception erronée de l’avis de son partenaire.

**Conclusions :** Les perceptions des femmes de l’avis de leurs conjoints sont un déterminant bien plus fort de l’utilisation contraceptive que l’opinion réelle de ces derniers, et stéréotyper l’opinion des hommes concernant la planification familiale est une erreur d’appréciation courante. Cependant, les résultats de recherche suggèrent aussi que ces malentendus pourraient servir la capacité des femmes à négocier leur utilisation de la contraception.

**Résumé simplifié**

La recherche indique que les femmes vivant en Afrique subsaharien peuvent ne pas utiliser de méthodes de contraception si leurs partenaires y sont opposés. Toutefois, la manière dont cette relation a été mesurée jusqu’à présent est marquée par des lacunes méthodologiques. Par exemple, la question de savoir si le partenaire est favorable à la contraception n’est souvent posée qu’aux femmes, et les enquêtes évaluent rarement comment les femmes savent que leurs partenaires sont opposés à la contraception and avec quelle force cela leur a été communiqué ; du reste, il est rarement demandé au partenaire concerné son opinion réelle du sujet.

Dans cette étude, nous tentons de répondre à certaines de ces questions en interviewant séparément des hommes et des femmes en couple et comparons leurs avis sur l’utilisation de la planification familiale. Cette recherche utilise une enquête basée sur la population menée auprès de couples vivant dans des camps militaires de la capitale de la République Démocratique du Congo, Kinshasa.

Les résultats montrent que les femmes ont généralement une faible connaissance de l’opinion réelle de leurs partenaires, mais agissent néanmoins en fonction de ces perceptions. En particulier, les femmes dont les conjoints sont opposés à la planification familiale mais qui perçoivent (à tort) un avis favorable sont parmi celles qui ont le plus de chances d’utiliser la contraception dans notre cohorte. Inversement, les femmes dont les perceptions correspondent au scénario de « faux négatif » (le conjoint est pour mais elle pense qu’il est contre) ont moins de chances d’utiliser la contraception moderne. Une analyse additionnelle indique que ce dernier scénario est plus répandu chez les femmes ayant un niveau d’études plus élevé que leurs conjoints, peut-être parce qu’elles tendraient à stéréotyper les désirs de planification familiale de leurs partenaires. Les résultats et l’analyse présentés ici soulèvent également la possibilité que les femmes pourraient in fine bénéficier de leur ignorance du souhait réel de leurs conjoints afin de réaliser leurs propres souhaits contraceptifs.

**Mots clés**

Contraception, hommes, approbation conjugale, communication au sein du couple, Afrique subsaharienne

## **Contexte**

L’implication des hommes dans le soutien de la décision et de l’utilisation contraceptive est un leitmotiv des programmes de planification familiale depuis la Conférence internationale sur la population et le développement de 1994 [1], avec des stratégies allant de changer les aspirations de fertilité masculine et les attitudes vis-à-vis de l’utilisation de la contraception [2], à augmenter l’autonomie des femmes dans la prise de décision liée à leur fécondité, [3] à améliorer la communication au sein du couple [4, 5, 6]. En Afrique subsaharienne, la supposition selon laquelle les hommes ont un désir « traditionnel » de famille nombreuse, et l’idée qu’ils jouent un rôle dominant dans la prise de décision au sein du ménage, les positionne comme des acteurs clés des interventions conçues pour améliorer l’utilisation contraceptive des femmes [7]. En effet, la recherche trouve de manière constante que l’approbation des hommes est associée à une plus grande utilisation de la contraception, indépendamment d’autres facteurs comme la perception des femmes de leur propre capacité à s’autodéterminer ou l’accessibilité perçue aux contraceptifs [8, 9, 10, 11, 12]. De plus, cet effet semble bidirectionnel puisque la désapprobation des hommes émerge dans certaines études comme le frein le plus puissant à l’utilisation de la planification familiale [13, 14, 15].

Le rôle clé de l’approbation de la contraception par les hommes est cependant complexifié car les données existantes suggèrent aussi fortement que les femmes tendent à avoir des perceptions inexactes de l’opinion réelle de leurs conjoints concernant leur utilisation de la planification familiale [16]. La faible validité de ce que disent les femmes mariées de l’approbation de la planification familiale par leurs conjoints a été attribuée au manque de communication au sein du couple [17] et à une tendance à stéréotyper le désir de fécondité de leurs partenaires (« Tous les hommes veulent une famille nombreuse ») [18]. Du point de vue des partenaires masculins, la croyance très répandue que l’homme doit avoir le dernier mot dans les affaires familiales, mais que les femmes sont néanmoins les seules responsables de gérer leurs grossesses [19], renforce davantage encore les possibilités de malentendus. Les études comparatives existantes du désir de fécondité des hommes et des femmes indiquent que la plupart de ces malentendus émanent typiquement d’une sous-estimation des femmes du désir de leurs conjoints de limiter la taille de leurs familles [15, 18], ou parce que les femmes associent le désir de leurs partenaires d’avoir plus d’enfants à une désapprobation de l’utilisation contraceptive [20]. Il en résulte une tendance au scénario « faux négatif », selon lequel les femmes perçoivent un avis défavorable à la contraception chez leurs partenaires, mais les enquêtes auprès des hommes suggèrent que la majorité d’entre eux soutiennent l’utilisation de la contraception, y compris dans des contextes à forte fécondité comme au Nigeria et en Éthiopie [21, 22].

Peu d’études existantes permettent de comparer la perception des femmes de l’approbation à l’opinion réelle de leurs partenaires masculins, et le fait de ne disposer que de la préférence perçue de ces partenaires plutôt que de leurs préférences réelles en tant que variable explicative est souvent citée comme une limite [12, 17, 23]. La recherche présentée ici permet de mieux comprendre ces malentendus au regard de l’utilisation contraceptive en comparant les réponses de couples ayant répondu individuellement aux mêmes questions. Les couples ont tous été recrutés dans les camps militaires dispersés dans tout Kinshasa, capitale de la République Démocratique du Congo (RDC). La RDC, l’un des pays les plus pauvres du monde, enregistre aussi l’un des taux de fécondité (6,0) les plus élevés et une prévalence contraceptive moderne parmi les plus faibles (14,2% en moyenne dans le pays et 24,5% à Kinshasa) au monde [24, 25]. En tant que pays cible de FP2020, la RDC, et les provinces de Kinshasa et Kongo Central en particulier, sont régulièrement ciblées par les enquêtes sur la population, et en 2016, les autorités militaires ont demandé à ce qu’une itération spécifique de l’enquête PMA2020 (*Performance Monitoring and Accountability 2020*) soit mise en œuvre dans les camps militaires de Kinshasa afin d’évaluer si ces populations avaient des besoins spécifiques, en comparaison à la population générale vivant dans les quartiers avoisinants. La portée plus restreinte de l’étude menée dans les camps militaires a permis aux chercheurs d’ajouter des modules thématiques spécifiques d’intérêt pour la population générale de RDC mais qui ne pouvaient typiquement pas être intégrés à l’enquête globale de PMA. Les résultats de la première vague de l’enquête ont indiqué, entre autres conclusions, que les femmes résidant dans les camps militaires avaient de forts besoins non satisfaits en matière de services de planification familiale [26]. Les données présentées dans le présent article proviennent de la deuxième vague de l’enquête PMA dans les camps militaires, mise en œuvre en janvier 2020. Cette vague vise à tracer les tendances contraceptives après quatre années de mise en œuvre de diverses interventions par les partenaires en planification familiale dans les camps militaires, mais comprend aussi des questions complémentaires sur les désirs de fécondité et l’approbation de la contraception, posées indépendamment à chacun des partenaires homme et femme de couples établis. L’objectif des analyses présentées ici est d’abord d’estimer la prévalence des perceptions exactes, les perceptions erronément positives et erronément négatives de l’approbation des hommes de l’utilisation de la contraception par leurs conjointes, et de comparer l’importance relative de l’approbation réelle et perçue au regard de l’utilisation contraceptive des femmes. Nous cherchons ensuite à déterminer les caractéristiques individuelles et des couples associées aux perceptions exactes et inexactes de l’avis du conjoint selon la femme, en portant une attention particulière aux « faux négatifs » et aux « faux positifs ».

## **Méthodes**

*Données*

La collecte des données auprès de la population des camps militaires a utilisé un modèle d’enquête par grappes en plusieurs étapes. Kinshasa compte 17 camps militaires, où résident environ 3% (305 000) de la population totale de la ville [26]. Les ménages dans ces camps tendent à avoir au moins un membre militaire en service actif, toutefois, des familles élargies sans affiliation directe à l’armée peuvent aussi habiter dans ces camps. Tous les résident(e)s de ces camps ont néanmoins accès aux formations sanitaires militaires du camps, y compris aux services de planification familiale offerts par le Programme Militaire de Santé de la Reproduction (PMSR).

Sur les 17 camps militaires de Kinshasa, 10 ont été sélectionnés au hasard selon la méthode d’échantillonnage proportionnelle à la taille de la population. Ces 10 camps militaires ont ensuite été divisés en zones de dénombrement (ZD) de taille similaire. Une ZD a ensuite été sélectionnée au hasard dans chacun des 10 camps. L’équipe de collecte des données était composée de femmes enquêtrices résidentes (ER). Toutes les ER étaient elles-mêmes résidentes des camps militaires, ont été formées aux aspects techniques et éthiques de l’enquête, et avaient participé aux vagues précédentes de l’enquête PMA en 2016. Elles ont d’abord recensé tous les ménages de la ZD sélectionnée. En 2016, 33 ménages avaient été sélectionnés au hasard dans chaque ZD. Puisque nous cherchions à interviewer des couples, nous n’avons interviewé que des femmes mariées ou en union qui habitaient avec leurs partenaires dans ces ménages. Les femmes divorcées, veuves ou n’ayant jamais été mariées (environ 2% de l’échantillon total) ont été exclues de notre analyse. Afin d’atteindre une taille d’échantillon comparable à celle que nous avions utilisée en 2016, tout en limitant notre population aux femmes mariées ou en union, nous avons ajouté au hasard 14 ménages dans chaque ZD , soit un total de 47 ménages. Dans la mesure où la taille de l’échantillon a été calculée en fonction des femmes, les femmes dans les ménages sélectionnés ont été interviewées d’abord, et leurs partenaires ensuite. Bien que certains hommes aient indiqué avoir une relation polygame, aucune des femmes interviewées dans notre échantillon n’était liée au même partenaire. Le taux de réponse à cette enquête est supérieur à 99%. Les hommes et les femmes au sein de chaque couple ont été interviewés par la même ER mais de manière individuelle, à un endroit de leur choix et dans des salles séparées pour garantir la confidentialité de l’entretien et le respect de leur vie privée. Les interviews ont été conduites en français ou dans le dialecte local dans lequel les répondant(e)s se sentaient le plus à l’aise (le plus souvent, en Lingala qui est la *lingua franca* de l’armée en RDC) et les réponses ont été enregistrées en français sur des smartphones utilisant l’application OpenDataKit. [26]

La collecte des données et le protocole de recherche ont été autorisés par le Comité d’éthique biomédicale (IRB) de l’Université de Tulane (étude 492318) et celui de l’École de Santé Publique de Kinshasa (#ESP/CE/070/2017).

Toutes les personnes ayant pris part à cette étude ont donné par écrit leur consentement informé à participer à l’enquête.

*Analyse*

Cette étude se focalise sur deux séries de variables de résultats : (1) les perceptions exactes ou inexactes de l’approbation des partenaires hommes de l’utilisation contraceptives chez les répondantes femmes, et (2) l’utilisation contraceptive moderne chez les répondantes femmes. La perception de l’approbation du partenaire homme est définie par la réponse de la femme à la question : « *Pensez-vous que votre partenaire est favorable/ serait favorable à ce que vous utilisiez des contraceptifs modernes* ? » Les modalités de réponse possibles étaient : « oui », « non », « cela dépend de la méthode » et « ne sait pas », que nous avons catégorisées comme un « avis perçu comme favorable » si la réponse était « oui », et « un avis perçu comme défavorable » pour toutes les autres réponses. Les mêmes catégories binaires ont été appliquées aux réponses des répondants à la question : *« Seriez-vous favorable à ce que votre partenaire utilise la contraception ? »*. (Les enquêtrices n’ont pas divulgué aux hommes si leurs conjointes avaient rapporté utiliser ou non la contraception). Toute autre réponse que « oui » a été enregistrée comme un avis défavorable. La comparaison des perceptions des femmes aux opinions réelles des hommes a mené à quatre scénarios principaux : La femme perçoit correctement un avis favorable (S1) ; La femme perçoit correctement un avis défavorable (S2) ; La femme perçoit erronément un avis défavorable (faux négatif) (S3) ; et La femme perçoit erronément un avis favorable (faux positif) (S4), dont les combinaisons ont mené à une « perception exacte de l’avis du partenaire » (S1+S2 = S5) et une « perception inexacte » (S3 + S4 = S6).

Nous avons d’abord mené une analyse bivariée pour tester l’association de ces scénarios avec l’utilisation contraceptive moderne du couple ; ensuite, pour tester l’association entre l’utilisation contraceptive moderne et les scénarios mentionnés après avoir contrôlé d’autres variables indépendantes influentes, nous avons inclus les caractéristiques de chaque personne et des couples dans un modèle de régression multiple.

Enfin, nous avons mené une autre série d’analyses par régression pour déterminer si certaines caractéristiques individuelles ou du couple étaient associées de manière significative aux scénarios de perception dont il s’avère qu’ils ont plus de chances d’augmenter ou de réduire l’utilisation contraceptive, y compris la perception inexacte de l’opinion du partenaire, l’approbation perçue, et la perception d’un faux négatif. Les variables indépendantes incluses dans toutes les régressions sont des facteurs dont on présume qu’ils influencent les relations de pouvoir au sein des couples et l’utilisation de la contraception, tels que l’âge de la femme, le niveau d’études, les différences de niveaux d’études dans le couple, le nombre d’enfants en vie, si chaque partenaire souhaite avoir plus d’enfants ou non, si chaque partenaire a parlé de son nombre d’enfants désiré, le nombre idéal d’enfants selon l’homme et son statut militaire (en service actif ou non).

## **Résultats**

L’échantillon de l’étude comprend 252 couples, mariés ou vivant en union. Comme l’illustre le Tableau 1, les femmes sont en moyenne plus jeunes et ont un niveau d’études légèrement inférieur à celui de leurs conjoints. Environ la moitié des hommes et seulement 2,9% des femmes sont en service militaire actif. La plupart des personnes enquêtées ont terminé au moins l’école secondaire (83,2% des femmes et 90,3% des hommes) et les niveaux d’études sont similaires dans 57,0% des couples, tandis qu’un quart des hommes (25,9%) ont un niveau d’études plus élevé que celui de leurs conjointes, et 17,1% des femmes ont suivi des études plus longues que leurs conjoints (Cf. Tableau 1 ci-dessous). Des écarts observés dans les réponses sur le statut conjugal, dont les hommes rapportent de manière disproportionnée être mariés et avoir un nombre légèrement plus élevé d’enfants en vie, sont probablement dus au fait que leur partenaire résidant dans le camp n’est pas toujours leur conjointe principale ou officielle. Indépendamment de cela, toutes les questions de planification familiale ont été posées en rapport à la/au partenaire avec qui l’enquêté(e) vivait dans le camp au moment de l’enquête, et cette personne est aussi celle qui a été interviewée en tant que « deuxième moitié » du couple.

Tableau 1. Caractéristiques démographiques des couples de l’étude (N=252)

|  | **Femmes (%)** | **Hommes (%)** |
| --- | --- | --- |
| **Age** | | |
| <20 | 8,6 | 1,2 |
| 20-24 | 15,6 | 4,9 |
| 25-29 | 18,2 | 7,9 |
| 30-34 | 20,9 | 15,3 |
| 35-39 | 16,0 | 15,2 |
| >=40 | 20,7 | 55,5 |
|  | | |
| **Niveau d’études** | |  |
| Jamais scolarisé(e)s/ École primaire | 16,8 | 9,7 |
| École secondaire | 64,6 | 68,8 |
| Études supérieures | 18,6 | 21,5 |
|  | | |
| **Différences de niveaux d’études** |  |  |
| Même niveau d’études | 57,0 | n/a |
| L’homme a un niveau d’études plus élevé | 25,9 | n/a |
| La femme a un niveau d’études plus élevé | 17,1 | n/a |
|  | | |
| **Statut de militaire d’active** | 2,9 | 56,2 |
|  | | |
| **Marié(e)** | 45,8 | 73,2 |
| **En union** | 54,0 | 26,8 |
|  |  |  |
| **Nombre moyen d’enfants en vie** | 2,5 | 2,9 |

Dans le Tableau 2, nous comparons l’avis favorable des hommes en couple concernant l’utilisation contraceptive à la perception des femmes de l’avis de leurs conjoints. Interrogés séparément, 59,8% des hommes déclarent être défavorables à ce que leurs conjointes utilisent la contraception, mais une plus grande proportion de femmes (73,4%) perçoit un avis défavorable. Lorsque les femmes ont une perception erronée de l’avis de leurs partenaires, elles ont tendance à croire plus souvent qu’ils sont opposés à la contraception alors qu’ils y sont en réalité favorables, plutôt que l’inverse : un quart (24,9%) des femmes pensent, à tort, que leurs partenaires ne soutiennent pas l’utilisation de la contraception (faux négatif). En revanche, 11,3% des femmes déclarent que leurs partenaires sont favorables à l’utilisation de la contraception alors qu’ils ne le sont en fait pas (faux positif). Au total, la perception de la femme de l’avis de son partenaire est juste dans 63,8% des cas. (*Cf. Tableau 2 ci-dessous*)

Tableau 2. Pourcentage des femmes qui perçoivent correctement ou incorrectement l’approbation ou désapprobation de leurs conjoints

|  | | **Approbation du partenaire homme** | | |
| --- | --- | --- | --- | --- |
|  |  | **Approuve (N)** | **Désapprouve (N)** | **Total (N)** |
| **Perception de la partenaire femme** | **Perçoit l’approbation** | 15,3% (47)  *S1* | 11,3% (32)  *S4* | **26,6% (79)** |
|  | **Perçoit la désapprobation** | 24,9% (61)  *S3* | 48,5% (112)  *S2* | **73,4% (173)** |
|  | **Total** | **40,2% (108)** | **59,8% (144)** | **100,0% (252)** |

Nous avons ensuite examiné l’association entre les scénarios du Tableau 2 (soit la combinaison de l’opinion réelle du partenaire et la perception de la femme d’un avis favorable ou défavorable de son conjoint) et l’utilisation contraceptive moderne dans le couple (Tableau 3). Comme on peut s’y attendre, l’utilisation contraceptive moderne est plus élevée (41,3%) lorsque l’homme y est favorable et que sa conjointe perçoit correctement son approbation, et elle est plus faible (19,4%) lorsque la femme perçoit correctement la désapprobation de son conjoint (p=0.1201).

Nos résultats indiquent cependant que l’utilisation contraceptive moderne est similaire, qu’elle soit élevée et faible, quelle que soit l’opinion réelle de l’homme, à condition que la femme la perçoive comme un avis favorable ou défavorable, et ces associations sont bien plus significatives. L’utilisation contraceptive moderne est de 40,3% (valeur-p < 0,05) chez les femmes qui pensent que leurs conjoints sont favorables à la contraception, et de 19,5% (valeur-p < 0,05) chez celles qui pensent que leurs conjoints y sont défavorables. En réalité, 38,7% des femmes qui perçoivent erronément que leurs conjoints sont favorables à la contraception (faux positif) utilisent la contraceptive moderne (valeur-p<0.1), soit seulement 2,6 points de pourcentage de moins que les femmes qui pensent à juste titre que leurs conjoints soutiennent leur utilisation d’une méthode de contraception moderne. À l’autre extrémité du spectre, les femmes qui perçoivent, à tort, que leurs conjoints sont opposés à l’utilisation contraceptive (faux négatif) utilisent moins souvent la contraception moderne, bien que cette relation ne soit pas significative.

Tableau 3. Association entre la perception de l’approbation du partenaire et l’utilisation de la contraception moderne

|  | Fréq. | Utilisation contraceptive moderne (%) | Valeur-p* |
| --- | --- | --- | --- |
| Partenaire homme approuve/ Femme perçoit un avis favorable (S1) | 47 | 41,3 | 0,0828 |
| **Femme perçoit un avis favorable**  **(Quelle que soit l’opinion réelle du partenaire)** | **79** | **40,2** | **0,0116** |
| Partenaire homme désapprouve/ Femme perçoit un avis favorable (Faux positif) (S4) | 32 | 38,7 | 0,0730 |
| Partenaire homme approuve (quelle que soit la perception de la femme) | 108 | 27,9 | 0,5541 |
| Partenaire homme désapprouve (quelle que soit la perception de la femme) | 144 | 23,1 | 0,5541 |
| Partenaire homme approuve/ Femme perçoit un avis défavorable (Faux négatif) (S3) | 61 | 19,6 | 0,1672 |
| **Femme perçoit un avis défavorable**  **(Quelle que soit l’opinion réelle du partenaire)** | **173** | **19,5** | **0,0116** |
| Partenaire homme désapprouve/ Femme perçoit un avis défavorable (S2) | 112 | 19,4 | 0,1201 |

Pour tester davantage l’association entre les scénarios approbation/perception et l’utilisation contraceptive moderne, après avoir contrôlé les caractéristiques sociodémographiques des hommes et des femmes, nous avons effectué une régression logistique multivariée sur l’utilisation contraceptive moderne. Les résultats de cette analyse confirment le rôle bien plus significatif de la perception qu’a une femme de l’approbation de son conjoint comme déterminant de l’utilisation contraceptive (Cf. Tableau 4). Lorsque leurs conjoints sont défavorables à l’utilisation de la contraception, les femmes qui perçoivent erronément un avis favorable ont 2,52 fois plus de chances d’utiliser une méthode de contraception moderne que les femmes qui interprètent correctement l’opinion de leurs partenaires, et la relation est fortement significative (valeur-p = 0,007). Inversement, les femmes qui perçoivent erronément un avis négatif chez leurs partenaires (faux négatif) semblent avoir légèrement moins tendance à utiliser la contraception moderne, mais cette relation n’est pas significative (OR = 0,92, valeur-p = 0,805).

Parmi les autres caractéristiques des femmes et des hommes, peu sont associées de manière significative à l’utilisation contraceptive moderne : le rapport de cotes pour l’utilisation contraceptive baisse avec l’âge et les femmes de plus de 40 ans ont significativement moins de chances d’utiliser une méthode moderne (OR = 0,21, valeur-p < 0,05). Les femmes dont le partenaire est en service militaire actif ont aussi beaucoup moins de chances d’utiliser une méthode moderne (OR = 0,44, valeur-p < 0,1). En revanche, les femmes qui ont quatre enfants en vie ou plus ont trois fois plus de chances d’utiliser une méthode moderne (OR = 3,01, valeur-p < 0,05). Les différences de niveaux d’études, les variables du désir de fécondité telles que le souhait de l’un des partenaires d’avoir plus d’enfants et le nombre idéal d’enfants ou le fait d’avoir parlé du nombre d’enfants désiré, ne sont pas associées de manière significative à l’utilisation contraceptive moderne.

Tableau 4. Association des variables individuelles et du couple avec l’utilisation contraceptive moderne

| Utilisation contraceptive moderne (0,1) | Rapport de cotes | Erreur Type | Valeur-P |
| --- | --- | --- | --- |
| **Approbation réelle et perçue de l’utilisation contraceptive** | | | |
| Homme désapprouve/ Femme perçoit un avis défavorable (S2) | Réf. |  |  |
| Homme approuve/ Femme perçoit un avis favorable (S1) | 1,93 | 1,04 | 0,256 |
| Homme désapprouve/ Femme perçoit un avis favorable (S4) | 2,52*** | 0,67 | 0,007 |
| Homme approuve/ Femme perçoit un avis défavorable (S3) | 0,92 | 0,31 | 0,805 |
|  |  |  |  |
| **Âge de la femme** |  |  |  |
| <20 | Réf. |  |  |
| 20-24 | 1,59 | 0,66 | 0,295 |
| 25-29 | 1.58 | 0,48 | 0,165 |
| 30-34 | 0,76 | 0,44 | 0,644 |
| 35-39 | 0,53 | 0,24 | 0,193 |
| >=40 | 0,21** | 0,12 | 0,027 |
|  |  |  |  |
| **Différences de niveaux d’études** | | |  |
| Même niveau d’études | Réf. |  |  |
| L’homme a un niveau d’étude plus élevé | 1,16 | 0,49 | 0,728 |
| La femme a un niveau d’étude plus élevé | 0,89 | 0,29 | 0,722 |
|  |  |  |  |
| **Nombre d’enfants en vie** | | |  |
| 0-1 | Réf. |  |  |
| 2-4 | 1,10 | 0,71 | 0,887 |
| >4 | 3,11** | 1,52 | 0,045 |
|  |  |  |  |
| **L’homme est militaire d’active** | |  |  |
| Non | Réf. |  |  |
| Oui | 0,44* | 0,19 | 0,092 |
|  |  |  |  |
| **L’homme veut plus d’enfants** |  |  |  |
| Non |  |  |  |
| Oui | 0,79 | 0,46 | 0,699 |
|  |  |  |  |
| **La femme veut plus d’enfants** |  |  |  |
| Non |  |  |  |
| Oui | 1,14 | 0,48 | 0,773 |
|  |  |  |  |
| **L’homme a parlé de son nombre idéal d’enfants à sa partenaire** | | | |
| Non | Réf. |  |  |
| Oui | 1,28 | 0,27 | 0,273 |
|  |  |  |  |
| **La femme a parlé de son nombre idéal d’enfants à son partenaire** | | | |
| Non | Réf. |  |  |
| Oui | 0,75 | 0,33 | 0,536 |
|  |  |  |  |
| **Taille de la famille idéale selon l’homme** | 1,05 | 0,06 | 0,389 |

Puisque la perception de l’approbation des partenaires est si significativement associée à l’utilisation contraceptive chez les femmes, il semble important de déterminer si certains profiles individuels ou de couples sont associés aux perceptions (in)exactes de l’approbation, et les perceptions de « faux positifs » et « faux négatifs ». Pour ce faire, nous avons introduit trois modèles de régression logistique (Cf. Tableau 5) testant les associations entres les caractéristiques des femmes et des couples et trois scénarios spécifiques. Nous avons choisi ces scénarios en fonction de la forte association trouvée dans le modèle illustré précédemment (Tableau 4) : (1) la femme perçoit erronément l’opinion de son partenaire (36,2% de notre échantillon), (2) la femme perçoit un avis favorable quelle que soit l’opinion de son partenaire (26,6% de notre échantillon), et (3) la femme perçoit erronément un avis défavorable chez son partenaire (faux négatif = 24,9% de notre échantillon). Dans la mesure où un faible nombre de femmes répondent au scénario « faux positif », nous n’avons pas mené d’analyse de régression multiple pour ce résultat.

Les résultats de cette régression indiquent que parmi toutes les caractéristiques comprises dans le modèle, le nombre d’enfants en vie est fortement associé à la perception d’un avis favorable à l’utilisation de la contraception : les femmes ayant 2 à 4 enfants et les femmes ayant plus de 4 enfants ont deux à cinq fois plus de chances de déclarer que leurs partenaires soutiennent leur utilisation de la contraception (OR = 2,81 et 6,62 respectivement, valeur-p < 0,05). C’est aussi le cas des femmes déclarant vouloir plus d’enfants (OR = 2,13, valeur-p<0.05)

En revanche, les différences de niveaux d’études sont négativement associées à la perception de l’approbation du partenaire : les femmes ayant fait de plus longues études que leurs conjoints ont significativement moins de chances de percevoir l’avis de leurs conjoints comme favorable (OR = 0,35, valeur-p < 0,05). Cette variable est aussi significativement associée à la perception inexacte d’un avis comme favorable (OR = 1,9 valeur-p < 0.05), et c’est encore plus vrai lorsque l’avis est perçu à tort comme étant défavorable : les femmes ayant un plus haut niveau d’études que leurs partenaires ont trois fois plus de chances de rapporter un « faux négatif » (OR = 2,84, valeur-p < 0,01).

Le fait d’avoir parlé du nombre d’enfants désiré réduit la probabilité des femmes de percevoir erronément l’avis de leurs partenaires comme favorable (OR = 0,69, valeur-p < 0,1), et la probabilité d’un « faux négatif » diminue significativement lorsque l’homme a communiqué à sa partenaire le nombre d’enfants qu’il souhaite avoir (OR = 0,27, valeur-p < 0,01).

Tableau 5. Analyse régressive logistique multivariée des perceptions exactes et inexactes de l’approbation du partenaire concernant l’utilisation contraceptive

|  | Perception inexacte | Avis perçu comme favorable | Avis perçu à tort comme défavorable  (faux négatif) |
| --- | --- | --- | --- |
| **Age de la femme** | | | |
| <20 | Réf. |  |  |
| 20-24 | 2,03 | 0,69 | 3,57 |
| 25-29 | 0,52 | 0,27 | 1,40 |
| 30-34 | 1,39 | 0,15* | 5,72 |
| 35-39 | 0,79 | 0,13 | 2,20 |
| >=40 | 0,72 | 0,08 | 2,01 |
| **Différences de niveaux d’études** | | | |
| Même niveau d’étude | Réf. |  |  |
| L’homme a un niveau d’études plus élevé | 1,64 | 1,73* | 1,14 |
| La femme a un niveau d’études plus élevé | 1,91** | 0,35** | 2,84*** |
| **Nombre d’enfants en vie** | | | |
| 0-1 | Réf. |  |  |
| 2-4 | 1,90 | 2,81** | 1,46 |
| >4 | 1,25 | 6,62** | 1,03 |
| **L’homme est militaire en service actif** | |  |  |
| Non | Réf. |  |  |
| Oui | 1,51 | 0,58 | 1,65 |
| **L’homme veut plus d’enfants** |  |  |  |
| Non | Réf. |  |  |
| Oui | 0,84 | 0,45 | 1,50 |
| **La femme veut plus d’enfants** |  |  |  |
| Non | Réf. |  |  |
| Oui | 1,15 | 2,13** | 0,95 |
| **L’homme a parlé du nombre d’enfants qu’il désire avec sa partenaire** | | | |
| Non | Réf. |  |  |
| Oui | 0,56 | 4,67* | 0,27*** |
|  | | | |
| Non | Réf. |  |  |
| Oui | 0,67 | 0,73 | 0,74 |
| **Taille de la famille idéale selon l’homme** | 0,93 | 0,99 | 0,92 |

Les différences de taille idéale de la famille entre l’homme et la femme, ainsi que le fait d’avoir déjà utilisé la contraception ou de l’utiliser actuellement, ont initialement été ajoutés au modèle mais n’ont généré aucun résultat significatif.

Dans la mesure où les différences de niveaux d’études et le fait d’avoir parlé de son nombre d’enfants désiré sont fortement associés à la perception (in)exacte de l’approbation du partenaire de l’utilisation contraceptive, nous avons mené une analyse séparée pour évaluer si le niveau d’étude est associé à un niveau de communication plus élevé sur les désirs de fécondité. (Tableau 6). Bien qu’un plus haut niveau d’études chez l’un(e) des partenaires soit associé à la probabilité d’avoir communiqué le nombre d’enfants désiré (valeur-p < 0,1), les différences de niveaux d’études n’ont pas de poids significatif sur ce résultat. Ainsi, les femmes ayant suivi des études plus longues que leurs conjoints n’ont ni plus ni moins de chances d’être plus informées sur le désir de fécondité de leurs partenaires.

Tableau 6. Association entre le niveau d’études individuel, les différences de niveaux d’études et la probabilité d’avoir parlé du nombre d’enfants désiré

|  | **L’homme a parlé du nombre d’enfants qu’il désire (%)** | |
| --- | --- | --- |
| **Niveau d’études de l’homme** |  | P=0,0883* |
| **Jamais scolarisé/École primaire** | 32,56 |  |
| **École secondaire** | 56,63 |  |
| **Études supérieures** | 74,94 |  |
|  |  |  |
|  | **La femme a parlé du nombre d’enfants qu’elle désire (%)** | |
| **Niveau d’études de la femme** |  | P=0.0815* |
| **Jamais scolarisée/ École primaire** | 57,26 |  |
| **École secondaire** | 61,66 |  |
| **Études supérieures** | 74,25 |  |
|  |  |  |
|  | **L’homme a parlé du nombre d’enfants qu’il désire** | |
| **Différences de niveaux d’études** |  | P = 0,4641* |
| **Même niveau d’études** | 62,88 |  |
| **L’homme a fait des étude plus longues** | 58,9 |  |
| **La femme a fait des études plus longues** | 41,8 |  |
|  |  |  |
|  | **La femme a parlé du nombre d’enfants qu’elle désire** | |
| **Différences de niveaux d’études** |  | P = 0,1990* |
| **Même niveau d’études** | 64,36 |  |
| **L’homme a fait des étude plus longues** | 57,35 |  |
| **La femme a fait des études plus longues** | 68,57 |  |

**Discussion**

Les résultats de cette recherche parmi les couples militaires résidant à Kinshasa confirment que les femmes tendent à avoir une faible connaissance de l’opinion de leurs partenaires concernant l’utilisation de la contraception : près de la moitié des femmes de notre échantillon ont des perceptions erronées, et la majorité perçoit de manière inexacte la position de leurs conjoints comme étant défavorable à la contraception (faux négatif), tandis qu’environ une femme sur dix rapporte un faux positif (percevant, à tort, un avis favorable chez leur partenaire). Le scénario du faux négatif, qui concerne 24,9% de notre échantillon, pourrait être le produit de stéréotypes en l’absence de dialogue sur la planification familiale entre les conjoints, comme cela a été observé dans d’autres environnements à forte fécondité et à faible utilisation contraceptive, où les femmes tendent à projeter sur leurs partenaires l’attitude présumée de la plupart des hommes vivant dans ces environnements [11, 21].

Ces erreurs d’appréciation de l’opinion des hommes sur la contraception sont préoccupantes car nos résultats indiquent que, indépendamment de l’approbation réelle des hommes de l’utilisation de la planification familiale, c’est la perception de leurs partenaires de cette opinion qui détermine la (non) utilisation, confirmant les résultats de recherches précédentes conduites en Afrique subsaharienne [11, 28]. En introduisant les variables individuelles et du couple dans les modèles, seules quelques caractéristiques attendues demeurent des prédicteurs indépendants de l’utilisation contraceptive (nombre d’enfants en vie) ou de la non-utilisation (femme de plus de 40 ans, conjoint engagé dans l’armée), mais la perception de l’approbation, particulièrement dans le cas des faux positifs, demeure un prédicateur indépendant puissant de l’utilisation contraceptive. Par conséquent, puisque près d’un quart de toutes les participantes rapportent une perception de « faux négatif », améliorer la communication sur le sujet au sein du couple pourrait augmenter l’utilisation de la contraception si l’avis favorable du partenaire est plus clairement communiqué.

Une analyse approfondie des variables associées aux perceptions inexactes, aux perceptions d’un avis favorable et aux « faux négatifs » confirme l’importance potentielle de la communication : les femmes ont quatre fois plus de chances de percevoir un avis favorable et quatre fois moins de chances d’avoir une perception négative erronée (faux négatif) si leur partenaire leur a parlé du nombre d’enfants qu’il souhaite avoir. Cependant, cette relation ne s’applique pas si la femme a exprimé son désir de fécondité, ce qui confirme le poids des préférences masculines dans les décisions relatives à la fécondité [29]. De manière intéressante, les femmes qui ont un grand nombre d’enfants en vie et celles qui déclarent vouloir plus d’enfants ont plus de chances de percevoir un avis favorable chez leurs partenaires. Cela suggère que les femmes qui ont réalisé leurs aspirations ou rempli les attentes de leurs conjoints en matière de fécondité, ou qui ont au moins l’intention de le faire, se sentent peut-être en droit de se reposer et présumeraient ainsi que leurs partenaires approuvent leur utilisation de la contraception pour espacer leurs prochaines grossesses.

Au-delà des désirs individuels et partagés de fécondité, et au-delà de la communication, le fait que la probabilité de percevoir un avis favorable à la planification familiale augmente lorsque les hommes ont un niveau d’études plus élevé que leurs partenaires renforce les données existantes suggérant que l’augmentation du niveau d’études des hommes joue un rôle important dans l’amélioration de la communication au sein du couple et les attitudes favorables vis-à-vis de la planification familiale [30, 31]. En revanche, le niveau d’études des femmes, et particulièrement les différences de niveaux d’études avec leurs partenaires, jouent un rôle relativement contre-intuitif dans nos résultats. Bien que les femmes ayant fait de plus longues études aient plus de chances d’avoir parlé du nombre d’enfants qu’elles souhaitent avoir avec leurs partenaires, les femmes ayant fait des études plus longues que leurs partenaires ont aussi beaucoup plus de chances de se tromper sur l’avis de leurs conjoints concernant la contraception, et cette erreur penche largement vers une perception erronée d’un avis défavorable. Cela pourrait être dû au fait que les femmes en couple avec des hommes d’un niveau d’études inférieur au leur sont plus enclines à stéréotyper les attitudes de leurs partenaires. Mais une autre possibilité est que les femmes avec un niveau d’études plus élevé que leurs conjoints pourraient déformer en toute connaissance de cause l’avis de leurs conjoints, en rapportant leur opposition, pour justifier le fait qu’elles n’utilisent pas de contraceptifs. Qu’elles aient elles-mêmes des désirs de forte fécondité, qu’elles aient peur des effets secondaires des contraceptifs modernes, ou qu’elles aient d’autres croyances négatives sur la contraception, les femmes avec un niveau d’études élevé sont plus susceptibles de comprendre que la désapprobation de leurs partenaires est une réponse plus acceptable pour justifier de leur non-utilisation de la contraception, car elles peuvent ainsi se dégager de la responsabilité décisionnelle. Les recherches conduites en Afrique subsaharienne indiquent de manière constante que les femmes tendent à avoir des attitudes plus conservatrices que les hommes [28], et que la relation inversée classique entre le niveau d’études des femmes et le nombre désiré d’enfants n’est pas aussi fortement marquée que dans d’autres contextes en voie de développement [31].

Cette hypothèse suggère que les décalages observés entre la perception de l’opinion des conjoints sur la contraception et l’opinion réelle de ces derniers pourraient avoir une certaine utilité. Les femmes qui, pour une raison ou pour une autre, ne souhaitent pas utiliser la contraception moderne ou préfèrent avoir plus d’enfants pourraient justifier leur décision de manière socialement acceptable en attribuant à leurs conjoints un avis défavorable à la planification familiale. Inversement, les femmes peuvent feindre de ne pas connaître l’opposition de leurs partenaires (faux positifs) pour éviter d’être accusées d’utiliser la contraception en cachette. Étant donné que la plupart des hommes de notre échantillon n’approuvent pas l’utilisation de la contraception, et compte tenu du poids de l’opinion perçue des hommes dans les décisions de planification familiale, améliorer la connaissance des femmes sur l’avis réel de leurs partenaires ne bénéficierait pas toujours à celles-ci. Wolff, Blanc et al., dans leur analyse du rôle de la négociation au sein du couple dans les besoins non satisfaits de contraception en Ouganda, ont noté de manière similaire que « lorsque des désaccords sérieux existent, une plus grande communication peut ne pas être un bien universel » [18], et d’autres études ont souligné les tensions des relations hommes-femmes, avec des répercussions parfois brutales sur l’agenda de santé des femmes, générées par des interventions conçues pour impliquer frontalement les hommes dans les décisions contraceptives sans d’abord tenir compte des paramètres culturels [32].

Cette étude de la dissonance entre l’approbation perçue et réelle de l’utilisation contraceptive au sein des couples effleure donc un sujet important pour les programmes de planification familiale qui s’efforcent d’améliorer l’engagement des hommes tout en préservant le pouvoir de décision des femmes et leur droit de choisir leur planification familiale : que les erreurs de perception des femmes soient honnêtes ou intentionnelles, ces malentendus pourraient à certains égards davantage servir les intentions contraceptives des femmes que ne le ferait une parfaite communication sur les décisions de planification familiale dans le couple.

Notre étude comporte néanmoins plusieurs limites, particulièrement en termes de biais de désirabilité. Les répondants de sexe masculin en particulier sont plus enclins à déclarer qu’ils seraient favorables à ce que leurs conjointes utilisent la contraception car ils savent que c’est la « bonne » réponse. De plus, nous avons utilisé la question « avez-vous déjà parlé du nombre d’enfants que vous voulez avoir ? » comme variable de communication sur la planification familiale, mais il n’est pas certain qu’un accord sur les désirs de fécondité se traduise adéquatement en accord sur l’utilisation de la contraception dans le couple. D’autre part, notre instrument d’enquête ne nous permet pas d’établir la fréquence, la durée et la qualité de ces conversations, autant de variables dont nous pouvons présumer qu’elles ont un impact sur la perception d’une femme de l’opinion de son conjoint. Nous avons également choisi l’utilisation contraceptive moderne, plutôt que l’intention d’utiliser la contraception, comme résultat clé rapporté en raison de sa plus grande fiabilité, mais ce choix repose sur le postulat que les femmes vivant dans les camps militaires font toutes face à des obstacles et des opportunités similaires pour accéder aux services de contraception. Enfin, les prochaines recherches sur le sujet devront explorer la mesure dans laquelle l’environnement militaire de l’étude a affecté nos résultats, et si ces derniers peuvent être généralisés à d’autres couples vivant à Kinshasa –d’autant que des besoins non satisfaits de planification familiale plus élevés et des relations hommes-femmes plus inégales dans ce contexte que dans la population générale ont déjà été enregistrés [26], [33] et la recherche récente indique que bien que des tendances contraceptives similaires semblent se dessiner dans la population générale et parmi les habitants des camps militaires à Kinshasa, ces derniers continuent d’afficher certaines spécificités dues à la forte mobilité des hommes en particulier [34].

**Conclusion**

Cette étude offre des données probantes solides sur le fait qu’indépendamment de l’opinion réelle des hommes sur la planification familiale, la perception qu’ont les femmes de l’avis favorable ou défavorable de leurs conjoints pèse beaucoup plus lourd sur leur utilisation de la contraception. Bien que les malentendus des femmes demeurent fréquents, particulièrement lorsqu’elles ont un niveau d’études plus élevé que leurs partenaires, les perceptions de « faux négatifs » et « faux positifs » pourraient avoir une certaine utilité en tant que telles dans les négociations sur la planification familiale. Tandis que les futures recherches et programmes devront chercher des manières efficaces d’impliquer davantage les hommes pour soutenir l’utilisation contraceptive, nos résultats suggèrent que, compte tenu des déséquilibres de pouvoir typiques dans la prise de décision en matière de procréation, la transparence absolue dans la communication au sein du couple pourrait aussi réduire la capacité de la femme à négocier ses propres choix de fécondité.

**List des abréviations**

CIPD Conférence internationale sur la population et le développement

ER Enquêtrice résidente

FP2020 Family Planning 2020

PMA Performance in Monitoring and Action

PMSR Programme Militaire pour la Santé de la Reproduction

RDC République Démocratique du Congo

ZD Zone de dénombrement

**Déclarations**

*Avis favorable du Comité d’éthique et consentement à participer*

La collecte des données auprès de la population des camps militaires a été autorisée par le Comité d’éthique de l’Université de Tulane (étude 492318) et celui de l’École de Santé Publique de Kinshasa (#ESP/CE/070/2017).

Toutes les personnes ayant pris part à cette étude ont donné par écrit leur consentement informé à participer à cette étude.

*Consentement pour publication*

Non applicable

*Disponibilité des données et des instruments d’enquête*

Les bases de données générées et/ou analysées pour cette étude ne sont pas publiquement disponibles pour des raisons de confidentialité et respect de la vie privée des répondant(e)s mais peuvent être raisonnablement sollicitées auprès de l’auteur(e) correspondant(e).

*Conflit d’intérêts*

Les auteurs déclarent n’avoir aucun conflit d’intérêt.

*Financement*

L’étude a été financée à travers l’accord de financement INV-007330 avec la Fondation Bill & Melinda Gates.

*Contributions des auteurs*

JHH a élaboré les questions de recherche, contribué à la supervision de la collecte des données sur le terrain et à l’analyse des données, et a développé le manuscrit. PA était responsable du modèle d’enquête général de PMA et de la méthodologie d’échantillonnage. PZA a contribué au protocole de l’étude et au développement de l’instrument d’enquête, et a dirigé les activités de collecte de données à Kinshasa. Tous les auteur(e)s ont lu et approuvé le manuscrit.

*Remerciements*

Les auteur(e)s souhaitent remercier les superviseurs de terrain de l’École de Santé Publique de Kinshasa, les enquêtrices résidentes qui ont conduit les enquêtes et tout(e)s les participant(e)s pour leurs contributions.

**Références**

[1] United Nations, Department of Economic and Social Affairs, United Nations Population Division. 1994 Report of the International Conference on Population and Development

[2] Piotrow, P. T., Kincaid, D. L., Hindin, M. J., Lettenmaier, C. L., Kuseka, I., Silberman, T., ... & Kim, Y. M. 1992. Changing men's attitudes and behavior: the Zimbabwe Male Motivation Project. *Studies in family planning*, *23*(6), 365-375.

[3] Do, M., & Kurimoto, N. 2012. Women's empowerment and choice of contraceptive methods in selected African countries. *International perspectives on sexual and reproductive health*, 23-33.

[4] Shattuck, D., Kerner, B., Gilles, K., Hartmann, M., Ng'ombe, T., & Guest, G. 2011. Encouraging contraceptive uptake by motivating men to communicate about family planning: the Malawi Male Motivator project. American journal of public health, 101(6), 1089-1095.

[5] Ogunjuyigbe, P. O., Ojofeitimi, E. O., & Liasu, A. 2009. Spousal communication, changes in partner attitude, and contraceptive use among the Yorubas of Southwest Nigeria. *Indian journal of community medicine: official publication of Indian Association of Preventive & Social Medicine*, *34*(2), 112.

[6] Hartmann, M., Gilles, K., Shattuck, D., Kerner, B., & Guest, G. 2012. Changes in couples' communication as a result of a male-involvement family planning intervention. *Journal of health communication*, *17*(7), 802-819.

[7] Vouking, M. Z., Evina, C. D., & Tadenfok, C. N. (2014). Male involvement in family planning decision making in sub-Saharan Africa- what the evidence suggests. *The Pan African medical journal*, *19*, 349. https://doi.org/10.11604/pamj.2014.19.349.5090

[8] Anasel, M. G., & Mlinga, U. J. 2014. Determinants of contraceptive use among married women in Tanzania: Policy implication. *African Population Studies*, *28*, 976-988.

[9] Mohammed, A., Woldeyohannes, D., Feleke, A., & Megabiaw, B. 2014. Determinants of modern contraceptive utilization among married women of reproductive age group in North Shoa Zone, Amhara Region, Ethiopia. *Reproductive health*, *11*(1), 1-7.

[10] Anguzu, R., Tweheyo, R., Sekandi, J. N., Zalwango, V., Muhumuza, C., Tusiime, S., & Serwadda, D. 2014. Knowledge and attitudes towards use of long-acting reversible contraceptives among women of reproductive age in Lubaga division, Kampala district, Uganda. *BMC research notes*, *7*(1), 1-9.

[11] Palamuleni ME. 2013. Socio-economic and demographic factors affecting contraceptive use in Malawi. Afr J Reprod Health; 17(3): 91–104.

[12] Tumlinson, K., Speizer, I. S., Davis, J. T., Fotso, J. C., Kuria, P., & Archer, L. H. 2013. Partner communication, discordant fertility goals, and contraceptive use in urban Kenya. African journal of reproductive health, 17(3), 79-90.

[13] Esber, A., Foraker, R. E., Hemed, M., & Norris, A. 2014. Partner approval and intention to use contraception among Zanzibari women presenting for post-abortion care. *Contraception*, *90*(1), 23-28

[14] Nwachukwu I and Obasi OO. 2008 Use of modern birth control methods among rural communities in Imo State, Nigeria. Afr J Reprod Health; 12(1): 101–108.

[15] Blackstone, S. R., Nwaozuru, U., & Iwelunmor, J. 2017. Factors influencing contraceptive use in sub-Saharan Africa: a systematic review. *International quarterly of community health education*, *37*(2), 79-91.

[16] Ijadunola MY, Abiona TC, Ijadunola KT, Afolabi OT, Esimai OA, OlaOlorun FM: Male involvement in family planning decision making in Ile-Ife, Osun State, Nigeria. Afr J Reprod Health. 2010, 14 (4 Spec no): 43-50.

[17] Prata, N., Bell, S., Fraser, A., Carvalho, A., & Neves, I. 2017. Partner support for family planning and modern contraceptive use in Luanda, Angola. *African journal of reproductive health*, *21*(1), 35-48.

[18] Wolff, B., Blanc, A. K., & Ssekamatte‐Ssebuliba, J. 2000. The role of couple negotiation in unmet need for contraception and the decision to stop childbearing in Uganda. Studies in family planning, 31(2), 124-137.

[19] Lusey H, San Sebastian M, Christianson M, Edin KE. 2017. Factors associated with gender equality among church-going young men in Kinshasa, Democratic Republic of Congo: a cross-sectional study. International journal for equity in health.;16(1):213. pmid:29228996

[20] Maharaj P and Cleland J. 2005. Women on top: the relative influence of wives and husbands on contraceptive use in KwaZulu-Natal, Women & Health, 41(2):31–41

[21] Orji EO. Onwudiegwu U. 2008. Contraceptive practice among married men in Nigeria. East Afr Med J; 7(80):357- 60.

[22] Bayray A. 2012. Assessment of male involvement in family planning use among men in southeastern zone of Tigray, Ethiopia. Scholarly Journal of Medicine. Vol 2(2): 1-10

[23] Khan, S., Mishra, V., Arnold, F., & Abderrahim, N. 2007. *Contraceptive Trends in Developing Countries. Calverton: ORC Macro*. DHS working papers, 16.

[24] INS, 2019. *Enquête par grappes à indicateurs multiples, 2017-2018, rapport de résultats de l’enquête* (Kinshasa, République Démocratique du Congo, 2019).

[25] Performance Monitoring for Action (PMA). 2020. *PMA Kinshasa 2020: Phase 1 Baseline Survey*. Baltimore, MD: Kinshasa School of Public Health, Tulane School of Public Health and Tropical Medicine, and Bill & Melinda Gates Institute for Population and Reproductive Health, Johns Hopkins Bloomberg School of Public Health. <https://www.pmadata.org/sites/default/files/data_product_results/DRC%20Kinshasa_Phase%201%20Results%20Brief_English_Final.pdf>, and *About PMA,* <https://www.pmadata.org>, accessed 1 March 2021

[26] Akilimali, P., Anglewicz, P., Engale, H. N., Kurhenga, G. K., Hernandez, J., Kayembe, P., & Bertrand, J. 2018. Differences in family planning outcomes between military and general populations in Kinshasa, Democratic Republic of the Congo: a cross-sectional analysis. *BMJ open*, *8*(12), e022295

[27] DeRose, L., Dodoo, F., Ezeh, A., & Owuor, T. (2004). Does Discussion of Family Planning Improve Knowledge of Partner's Attitude toward Contraceptives? *International Family Planning Perspectives,* *30*(2), 87-93. Retrieved August 27, 2021, from http://www.jstor.org/stable/3181031

[28] Nanda, G., Schuler, S. R., & Lenzi, R. 2013. The influence of gender attitudes on contraceptive use in Tanzania: New evidence using husband’s and wives’ survey data. *Journal of biosocial science*, *45*(3), 331-344.

[29] Iklaki, C. U., Ekabua, J. E., Abasiattai, A., Bassey, E. A., & Itam, I. H. (2005). Spousal communication in contraceptive decisions among antenatal patients in Calabar, Nigeria. *Nigerian Journal of Medicine*, *14*(4), 405-407.

[30] Kasongo, D. Y. 1993. Spouses' sociodemographic characteristics and contraceptive use in Kinshasa, Zaïre. *African Population Studies*, *1993*(8), 58-72

[31] Uchudi, J. M. (2001). Spouse’s socioeconomic characteristics and fertility differences in Sub-Saharan Africa: Does spouse’s education matter? *Journal of biosocial science*, *33*(4), 481.

[32] Bawah, A.A., Akweongo, P., Simmons, R., and Phillips, J.F. (1999), Women's Fears and Men's Anxieties: The Impact of Family Planning on Gender Relations in Northern Ghana. Studies in Family Planning, 30: 54-66. <https://doi.org/10.1111/j.1728-4465.1999.00054.x>

[33] Abdulrazaq, A. G., Kabir, S., Mohammad, N. S., & Suleiman, I. H. 2014. The effect of educational intervention on family planning knowledge, attitudes, and practices among married women in a military barrack in northern Nigeria. *African journal of reproductive health*, *18*(1), 93-101.

[34] Akilimali, P. Z., Nzuka, H. E., LaNasa, K. H., Wumba, A. M., Kayembe, P., Wisniewski, J., & Bertrand, J. T. (2021). The gap in contraceptive knowledge and use between the military and non-military populations of Kinshasa, DRC, 2016–2019. *PloS one*, *16*(7), e0254915
